# Batter’s Shoulder: Clinical Outcomes and Return to Sport

**DOI:** 10.7759/cureus.7681

**Published:** 2020-04-15

**Authors:** Kevin J OKeefe, Edward Haupt, William C Thomas, Joseph King, Michael Moser, Kevin W Farmer, Bradley Schoch

**Affiliations:** 1 Orthopaedics and Rehabilitation, University of Florida, Gainesville, USA; 2 Orthopaedics, Mayo Clinic, Jacksonville, USA

**Keywords:** shoulder, posterior labral tear, shoulder instability, batter's shoulder

## Abstract

Background

Batter’s shoulder has been defined as an acute posterior subluxation of the lead shoulder during a baseball swing causing a traumatic tear of the posterior labrum. There are limited data correlating repair techniques with return-to-play information but none utilizing standardized outcome measures. The purpose of this study is to examine a case series of patients for postoperative return-to-play and obtain follow-up using standardized outcome measures.

Methods

We retrospectively identified 10 patients with a batter’s shoulder injury. Patients were included if they met the criteria for batter’s shoulder injury. We attempted contact via telephone to complete Western Ontario Shoulder Instability (WOSI) and Disability of Arm Shoulder and Hand (QuickDASH) evaluations. We successfully reached five of the patients. The minimum follow-up was one year and the maximum was 11 years.

Results

All five patients in our cohort were able to return to play at the previous level without limitation. Patients reported a very low percentage limitation on the WOSI and QuickDASH questionnaires and results are detailed further on. Range of motion (ROM) and strength were not affected.

Conclusion

Batter’s shoulder is an infrequent cause of posterior labral tearing, leading to a painful swing that can limit sports activity. In our limited series, all patients treated with arthroscopic repair were able to return to play at the previous level, confirming a significantly improved prognosis for a batter’s shoulder injury in contrast to return to play after other causes of posterior labral tears.

## Introduction

Batter’s shoulder was originally described by Phillips and Andrews as an injury that results from an acute posterior instability event during a baseball swing (Paper: Philips BB, Andrews JR, Fleisig GS. Batter’s Shoulder: Posterior Instability of the Lead Shoulder, a Biomechanical Evaluation. Alabama Sports Medicine and Orthopaedic Center, Birmingham, AL. 2000). The injury can also occur in other athletes involved in sports requiring a forceful swing motion [1]. The injury scenario always involves the lead arm and results in posterior subluxation/dislocation with spontaneous reduction. This can damage the posterior labrum, predisposing patients to recurrent subluxation events and persistent pain. While it remains relatively uncommon, this injury can be disabling and result in loss of competitive function.

Posterior shoulder instability accounts for only 2% to 12% of patients with shoulder instability, making it far less common than anterior instability [2]. Most commonly, posterior instability is the result of repetitive posteriorly directed loads applied to the arm in forward flexion, adduction, and internal rotation, leading to attritional laxity of the posterior capsule [2-5]. Unlike attritional posterior labral tears, a batter's shoulder is caused by a single acute trauma resulting in a pathologic posterior labral tear.

Batter’s shoulder is believed to be caused by rotational forces and torsional stress on the posterior shoulder restraints (Philips BB, 2000). Significant energy is imparted into the ball during a baseball or golf-swing with rotational velocities exceeding 900 degrees/second [6-7]. These forces are further magnified when the bat or golf club fails to strike the ball, resulting in a lack of counterforce to the dynamic posterior pulling force, causing an increase in shear force across the glenohumeral joint [8]. After the initial injury, the resulting symptoms are often vague posterior shoulder pain during swinging or a marked decrease in power generated during the swing (Philips BB, 2000).

Return to play for athletes with traditional posterior labrocapsular instability has been reported between 55% and 71% with failure rates as high as 40% [9-11]. Return to play after batter’s shoulder is less well-known but has been cited at 75%-91% [8]. Most of the previous studies are over 10 years old and may not represent modern repair techniques currently being employed. The purpose of this study is to report the clinical outcomes, complications, and return to play in a series of patients treated for batter’s shoulder using modern labral repair techniques.

## Materials and methods

**Figure 1:**
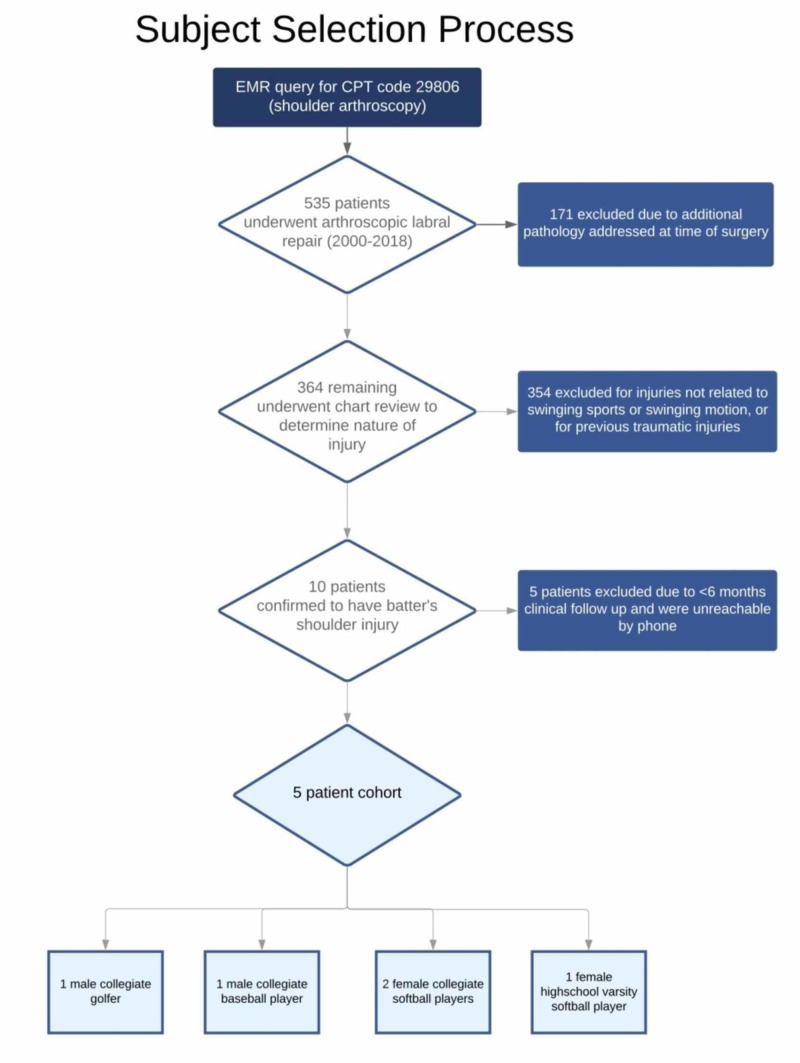
Subject Selection Process

A retrospective review was conducted of all shoulders treated with arthroscopic labral repair (Current Procedural Terminology (CPT) code 29806)) from 2000 to 2018. An electronic medical record query yielded 535 patients based on this single CPT code. Shoulders with multiple procedure codes indicating surgical treatment in excess of the labrum were excluded (171 shoulders). The electronic medical records of the remaining 364 shoulders were then reviewed to confirm the clinical diagnosis of a batter’s shoulder. Three-hundred fifty-four shoulders were confirmed to have injuries not related to a swinging sport or event, including patients with previous traumatic injuries to the shoulder or injuries from another sport. The remaining 10 patients were confirmed to have sustained a batter’s shoulder (acute instability or subluxation during swinging sports) based on a review of the medical history. Five of these patients did not have documented follow-up greater than six months and could not be reached by telephone and were excluded. The study cohort included three females and two males. All three females were competitive softball players (two collegiate, one high school varsity level). Of the males, one was a collegiate-level baseball player and one was a collegiate-level golfer (See Figure 1 for a visual summary). The average age at the time of injury was 20.4 years (range 17-23 years). The lead shoulder was involved in all cases. All identified patients experienced pain localized to the posterior shoulder, which occurred most significantly during the swinging motion. The minimum follow-up was one year and the maximum was 11 years.

Demographic data obtained from the medical record included: age, gender, position, level of competition, lead batting arm, hand dominance, and length of follow-up. Clinical notes were reviewed to confirm the diagnosis, postoperative course, and eventual return to play.

**Figure 2:**
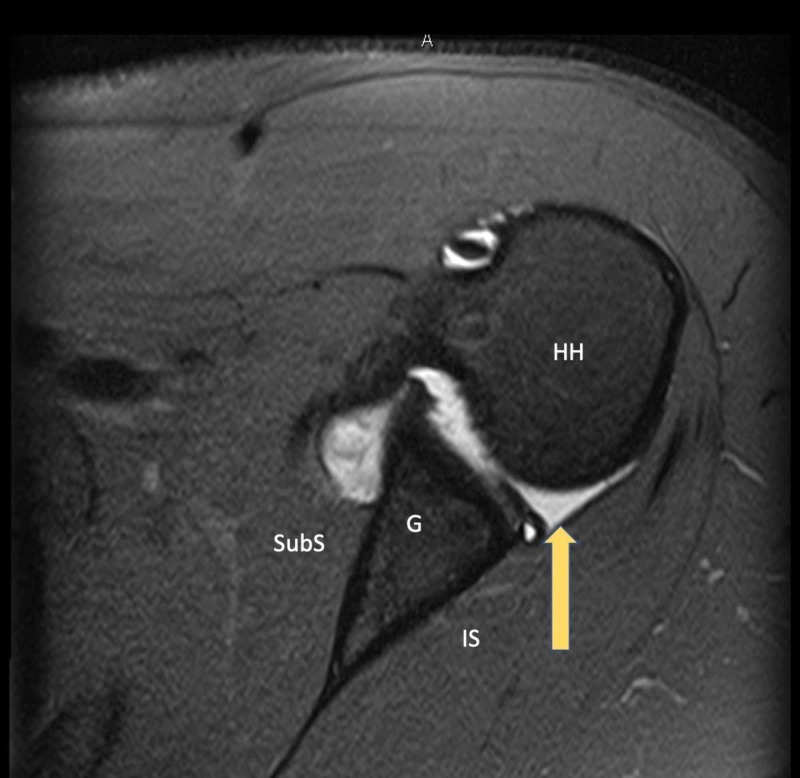
Magnetic Resonance Imaging (MRI) Demonstrating a Posterior Labral Tear Axial plane T2 fat-saturated image demonstrating a displaced posterior labral tear in a symptomatic athlete with a batter’s shoulder injury. Arrow - Posterior labral tear HH - Humeral head; G - Glenoid; SubS - Subscapularis; IS - Infraspinatus

**Figure 3:**
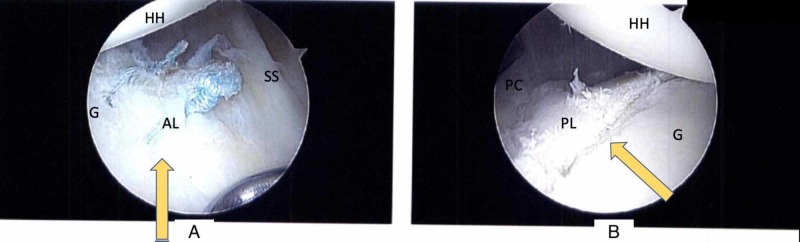
Intraoperative Identification 30-degree arthroscope viewing from the posterolateral portal of the right shoulder in lateral decubitus positioning, demonstrating recognition of a posterior labral “batter’s shoulder” tear intraoperatively in an athlete with a coincident anterior labral tear undergoing repair. A - Torn anterior labrum (arrow) after suture repair B - Torn posterior labrum (arrow) in the same patient HH - Humeral head; G - Glenoid; SS - Subscapularis; AL - Anterior labrum; PL - Posterior labrum; PC - Posterior capsule

Preoperative radiographic evaluation included Grashey and axillary lateral radiographs of the affected shoulder as well as MRI (with and without contrast). None of the five patients demonstrated bony changes of the glenoid or humeral head on plain radiographs. All MRIs were read by fellowship-trained musculoskeletal radiologists. Four of the five shoulders demonstrated an isolated posterior labral tear (Figure 2), and the remaining patient was taken to the operating room based on clinical suspicion and confirmed to have an isolated posterior labral tear intraoperatively (Figure 3).

Data extracted from the operative report included indication for surgery and surgery performed with specific regard to technique and implants utilized. Indications for surgery included: (1) failure of conservative therapy, including physical therapy and activity modification; (2) inability to return to the previous level of play. This was a single-institution, multi-surgeon cohort. All patients in this cohort underwent arthroscopic labral repair with suture-anchor capsulolabral fixation to the glenoid rim.

Postoperatively, patients were placed in a simple sling. Passive ROM in the scapular plane to 90 degrees, pendulums, and Codman exercises was initiated at the initial postoperative visit on postoperative Day 1 or 2. Internal rotation was limited to zero degrees for four weeks and then gradually advanced. The sling was discontinued at four weeks and active ROM initiated. Isotonic strengthening started eight weeks postoperatively. Patients were permitted to do dry swings at four months and then return to full batting at six months.

We reviewed postoperative follow-up records to ascertain patients’ final range of motion and return to sports as well as document postoperative complications. Four patients were also able to be reached for telephonic follow-up. These patients completed both Western Ontario Shoulder Instability (WOSI) and quickDASH (Disability Arm, Shoulder, Hand) scores [12-13].

## Results

All five shoulders were able to return to the previous level of play. No postoperative complications were observed, and no shoulder required reoperation for any reason. By the six-month follow-up visit, all five patients had regained 5 out of 5 strength in forward flexion, abduction, internal rotation, and external rotation, with no pain, and full return to pre-operative ROM. A summary of the following data is available in Tables 1-2. Four patients completed phone surveys at an average of 5.8 years from surgery (range 1-11). WOSI scores are typically reported by the patient making a mark on a 10 cm line, representing a percentage of limitation in various activities, ranging from 0% to 100%. We modified this to obtain the data via phone calls by having the patients identify verbally their percentage (0%-100%) of limitation in various activities. Questions are further divided into sub-score categories. WOSI scores showed an overall average score of 5.6% limitation (range 3.8%-8.6%). When broken down into subcategories, the physical symptoms sub-score showed an average of 4.75% and the sports/recreation sub-score showed an average of 1.8%.

**Table 1:** Survey Responses: WOSI WOSI: Western Ontario Shoulder Instability

		WOSI Score (% limitation)				
Name	Length of Follow-up	Physical symptoms	Sports/Rec/Work	Lifestyle	Emotional	Overall Score
Patient 1	1.1 years	10%	2.50%	17.50%	0%	8.60%
Patient 2	2 years	2%	0%	12.50%	3.30%	3.80%
Patient 3	11 years	2%	2.50%	7.50%	10%	4.30%
Patient 4	9 years	5%	2.50%	7.50%	10%	5.70%
	AVG	4.75%	1.88%	11.30%	5.80%	5.60%

**Table 2:** Survey Responses: QuickDASH QuickDASH: Disability Arm, Shoulder, Hand

		QuickDASH (% limitation)		
Name	Length of Follow-up	QuickDASH	QuickDASH Work	QuickDASH Sport
Patient 1	1.1 years	9.10%	0%	6.30%
Patient 2	2 years	9.10%	0%	0%
Patient 3	11 years	2.30%	0%	0%
Patient 4	9 years	0%	0%	0%

The quickDASH results are also reported as a percentage of limitation or disability in a given activity from 0% to 100%. We also used the extended quickDASH Sport and Work modules. The results from each individual patient can be seen in Tables 1-2. Patients reported less than a 10% limitation in any category, with most reporting a 0% limitation in the majority of categories. The Sport module showed minimal limitation for the majority of patients. The work module subset showed a 0% limitation among all four participants. The patient who was not reached by phone had adequate clinical follow-up for greater than two years. Additionally, he has continued his baseball career and is currently playing major league baseball without the need for additional shoulder operations.

## Discussion

Our goal was to perform a case series of batter’s shoulder patients to determine their rate of return to play, postoperative clinical data, and obtain longer-term follow-up regarding these parameters. Of the five studied patients, all returned to the preoperative range of motion and strength. All five returned to play. Our overall return-to-play rate seems to indicate a better prognosis regarding this specific injury as compared to a cohort of all posterior labral injuries.

Our data suggest that shoulder function and patient outcomes return to normal in our study population. These findings are comparable to previous papers specifically regarding batter’s shoulder [8]. Our study provides continued data regarding return to play but with the utilization of validated patient-reported outcome measures, which has not been attempted previously. Our data demonstrate that outcomes are favorable with a posterior labral tear due to a batter’s shoulder injury, possibly differentiating this entity from the larger group of posterior labral tears. Further studies are needed in this regard.

This study remains limited by its small size and retrospective design. However, due to the rarity of this condition, these limitations are difficult to avoid. Regardless, it is important to add to the limited literature in order to inform surgeons how this condition differs from more traditional attritional posterior labral tears. Additionally, using surgical CPT codes as the method of identifying the initial list of patients resulted in the exclusion of any patients with this injury that were treated nonoperatively. It remains unclear how responsive this condition is to traditional nonoperative management, as all patients in this cohort received surgery due to failing nonoperative management.

## Conclusions

Batter’s shoulder is an infrequent cause of posterior labral tearing leading to a painful swing that can limit sports activity. In our limited series, all patients treated with arthroscopic repair were able to return to play at the same level. This confirms an improved prognosis for posterior labral tear due to a batter’s shoulder injury as compared to other mechanisms of posterior labral tear.
